# Recent Progress on the Factorization Method for Electrical Impedance Tomography

**DOI:** 10.1155/2013/425184

**Published:** 2013-08-27

**Authors:** Bastian Harrach

**Affiliations:** Department of Mathematics, University of Würzburg, 97074 Würzburg, Germany

## Abstract

The Factorization Method is a noniterative method to detect the shape and position of conductivity anomalies inside an object. The method was introduced by Kirsch for inverse scattering problems and extended to electrical impedance tomography (EIT) by Brühl and Hanke. Since these pioneering works, substantial progress has been made on the theoretical foundations of the method. The necessary assumptions have been weakened, and the proofs have been considerably simplified. In this work, we aim to summarize this progress and present a state-of-the-art formulation of the Factorization Method for EIT with continuous data. In particular, we formulate the method for general piecewise analytic conductivities and give short and self-contained proofs.

## 1. Introduction

Electrical impedance tomography (EIT) aims to reconstruct the spatial conductivity distribution inside an imaging subject *Ω*⊆ℝ^*n*^ from current-voltage measurements on a part of its surface Σ⊆∂*Ω*. Mathematically, this leads to the problem of recovering the coefficient *σ*(*x*) in the elliptic partial differential equation

(1) $$\begin{array}{c} \nabla \cdot \sigma \nabla u_{\sigma }^{g}=0\;\text{in}\;\Omega ,\;\;\sigma \partial_{\nu }u_{\sigma }^{g}{|}_{\partial \Omega }=\{\begin{array}{cc} g & \mathrm{on}\;\;\Sigma ,\\ 0 & \text{else} \end{array} \end{array}$$

from knowledge of the corresponding Neumann-to-Dirichlet operator (NtD)

(2) $$\begin{array}{c} \Lambda \left(\sigma \right):g\mapsto u_{\sigma }^{g}{|}_{\Sigma }, \end{array}$$

where *u*_*σ*_^*g*^ is the solution of (1). We describe the precise mathematical setting in Section 2.1.

In several applications, EIT is used to determine the position of conductivity changes. This includes anomaly detection problems, where Λ(*σ*) is compared to a reference NtD Λ(*σ*_0_) in order to determine, if and where *σ* differs from a known background conductivity *σ*_0_. This problem also appears in time-difference EIT, where measurements at different times are compared to monitor temporal conductivity changes. These applications lead to the *shape reconstruction* problem of determining the support of *σ* − *σ*_0_ from Λ(*σ*) and Λ(*σ*_0_).

A prominent noniterative shape reconstruction method is the Factorization Method. It was introduced by Kirsch [1] for inverse scattering problems and extended to EIT by Brühl and Hanke [2–4]. In its original form (cf. [4]), the method assumes that

(3) $$\begin{array}{c} \sigma_{0}\left(x\right)=1,\;\;\sigma \left(x\right)=1+\kappa \left(x\right)\chi_{D}\left(x\right), \end{array}$$

where *D*⊆*Ω* is a union of separated, smoothly bounded, and simply connected domains, on which there is a conductivity jump of at least *ϵ* > 0; that is,

(4) $$\begin{array}{c} \kappa \left(x\right)\geq \epsilon \;\forall x\in D,\;\text{or}\;\;\kappa \left(x\right)\leq -\epsilon \;\forall x\in D. \end{array}$$

The method then characterizes the unknown shape *D* by a *range criterion*. For all unit vectors *d* ∈ ℝ^*n*^, ||*d*|| = 1,

(5) $$\begin{array}{c} z\in D\;\text{iff}\;\;\Phi_{z,d}{|}_{\Sigma }\in ℛ\left({\left|\Lambda \left(\sigma \right)-\Lambda \left(1\right)\right|}^{1/2}\right), \end{array}$$

where Φ_*z*,*d*_ is the so-called *dipole function*, that is, the solution of

(6) $$\begin{array}{c} \Delta \Phi_{z,d}=d\cdot \nabla \delta_{z}\;\text{in}\;\Omega ,\;\;\partial_{\nu }\Phi_{z,d}{|}_{\partial \Omega }=0. \end{array}$$

The range criterion (5) can be implemented numerically, so that each point *z* ∈ *Ω* can be tested whether it belongs to the unknown inclusion or not.

Substantial progress has been made on the Factorization Method since the original works of Kirsch, Brühl, and Hanke. In the following, we restrict ourselves to progress in the context of EIT. Overviews on the FM for EIT have been given by Hanke and Brühl [5], in the book of Kirsch and Grinberg [6], and in a recent chapter of Hanke and Kirsch in Scherzer's Handbook of Mathematical Methods in Imaging [7]. The FM for EIT has been treated as a special case of more general elliptic problems by Kirsch [8], the author [9], and by Nachman, Päivärinta and Teirilä [10]. A half-space setting has been considered by Hanke and Schappel [11]. Electrode models have been covered in the works of Brühl, Lechleiter, Hakula, Hanke, Hyvönen and Pursiainen [5, 12–15]. The FM has been extended to the complex conductivity case arising in frequency-difference EIT by Seo, Woo and the author [16, 17]. A priori separated indefinite inclusions have been treated by Schmitt [18], and Schmitt and Kirsch discussed the determination of the contrast level in the context of the FM for EIT in [19]. Hyvönen and the author have removed the assumptions on the inclusion contrast and boundary regularity in [20], and [21] discusses the relation of the FM to localized potentials.

In this work, we aim to summarize the theoretical progress and present a state-of-the-art formulation of the Factorization Method for EIT with continuous data. In particular, we will formulate the method for general piecewise analytic conductivities and give short and self-contained proofs.

## 2. Setting and Auxiliary Results

### 2.1. The Setting

We start by making the mathematical setting precise. Let *Ω*⊆ℝ^*n*^, *n* ≥ 2, denote a bounded domain with smooth boundary ∂*Ω* and outer normal vector *ν*. Let Σ⊆∂*Ω* be an open part of the boundary. *L*_+_^*∞*^(*Ω*) denotes the subspace of *L* ^*∞*^(*Ω*)-functions with positive essential infima. *H*_⋄_^1^(*Ω*) and *L*_⋄_^2^(Σ) denote the spaces of *H* ^1^- and *L* ^2^-functions with vanishing integral mean on ∂*Ω* (resp., Σ).

For *σ* ∈ *L*_+_^*∞*^(*Ω*) and *g* ∈ *L*_⋄_^2^(Σ), there exists a unique solution *u*_*σ*_^*g*^ ∈ *H*_⋄_^1^(*Ω*) of the elliptic partial differential equation

(7) $$\begin{array}{c} \nabla \cdot \sigma \nabla u_{\sigma }^{g}=0\;\text{in}\;\Omega ,\;\;\sigma \partial_{\nu }u_{\sigma }^{g}{|}_{\partial \Omega }=\{\begin{array}{cc} g & \mathrm{on}\;\Sigma ,\\ 0 & \text{else}, \end{array} \end{array}$$

so that we can define the Neumann-to-Dirichlet operator (NtD)

(8) $$\begin{array}{c} \Lambda \left(\sigma \right):L_{⋄}^{2}\left(\Sigma \right)\to L_{⋄}^{2}\left(\Sigma \right),\;\;g\mapsto u_{\sigma }^{g}{|}_{\Sigma }, \end{array}$$

where *u*_*σ*_^*g*^ ∈ *H*_⋄_^1^(*Ω*) solves (7). Λ(*σ*) is a self-adjoint, compact linear operator.

Let *σ*_0_ ∈ *L*_+_^*∞*^(*Ω*) be piecewise analytic. For each point *z* ∈ *Ω* that has a neighborhood in which *σ*_0_ is analytic, and each unit vector *d* ∈ ℝ^*n*^, ||*d*|| = 1, let Φ_*z*,*d*_ be the solution of

(9) $$\begin{array}{c} \nabla \cdot \sigma_{0}\nabla \Phi_{z,d}=d\cdot \nabla \delta_{z}\;\text{in}\;\;\Omega ,\;\;\sigma_{0}\partial_{\nu }\Phi_{z,d}{|}_{\partial \Omega }=0. \end{array}$$

Φ_*z*,*d*_ is called a *dipole function*.

### 2.2. Auxiliary Results

Our presentation of the Factorization Method in the next section relies on the following four lemmas. The first lemma is frequently called a *monotony lemma* since it shows that a larger conductivity leads to a smaller NtD. More precisely, it shows a relation between the difference of two NtDs and the difference of the corresponding conductivities and the interior energy of an electric potential. The second lemma shows that this energy term is the image of the adjoint of an auxiliary virtual measurement operator that is defined on a subregion of *Ω*. The third lemma is a functional analytic relation between the norm of an image of an operator and the range of its adjoint. Together with the first two lemmas, it implies that the range of the auxiliary virtual measurement operator can be calculated from the NtDs. Finally, using the previous dipole functions, the last lemma shows that the range of the auxiliary virtual measurement operator determines the region on which they are defined.

We start with the monotony lemma.

**Lemma 1**

Let *σ*_1_, *σ*_0_ ∈ *L*_+_^*∞*^(*Ω*). Then, for all *g* ∈ *L*_⋄_^2^(∂*Ω*),

(10) ∫Ω(σ0−σ1)|∇u0|2dx≤∫Σg(Λ1−Λ0)gds≤∫Ωσ0σ1(σ0−σ1)|∇u0|2dx,

where we abbreviated Λ_*j*_ : = Λ(*σ*_*j*_), *j* = 0,1, and *u*_0_ : = *u*_*σ*_0__^*g*^.

**Proof**

The lemma seems to go back to Ikehata, Kang, Seo, and Sheen [22, 23], cf. also the similar arguments in Kirsch [8], Ide et al. [24], and in the works of Seo and the author [16, 25]. For the sake of completeness, we copy the short proof from [25]. For all *g* ∈ *L*_⋄_^2^(∂*Ω*), we have that

(11) ∫Ωσ1∇u1·∇u0dx=∫Σgu0ds=∫Ωσ0∇u0·∇u0dx=∫ΣgΛ0gds.

Hence, from

(12) ∫Ωσ1∇(u1−u0)·∇(u1−u0)dx =∫Ωσ1|∇u1|2dx−∫Ωσ0|∇u0|2dx  +∫Ω(σ1−σ0)|∇u0|2dx,

we obtain that

(13) ∫Σg(Λ1−Λ0)gds=∫Ω(σ0−σ1)|∇u0|2dx +∫Ωσ1|∇(u1−u0)|2dx,

which already yields the first asserted inequality.

By interchanging *σ*_1_ and *σ*_0_, we conclude that

(14) ∫Σg(Λ0−Λ1)gds =∫Ω(σ1−σ0)|∇u1|2dx+∫Ωσ0|∇(u0−u1)|2dx =∫Ω(σ1|∇u1−σ0σ1∇u0|2+(σ0−σ02σ1)|∇u0|2)dx

and, hence, obtain the second inequality.

Given a reference conductivity *σ*_0_ ∈ *L*_+_^*∞*^(*Ω*) and a measurable subset *D*⊆*Ω*, we define the *virtual measurement operator L*_*D*_ by

(15) $$\begin{array}{c} L_{D}:L^{2}{\left(D\right)}^{n}\to L_{⋄}^{2}\left(\Sigma \right),\;\;F\mapsto v{|}_{\Sigma }, \end{array}$$

where *v* ∈ *H*_⋄_^1^(*Ω*) solves

(16) $$\begin{array}{c} \int_{\Omega }^{}\sigma_{0}\nabla v\cdot \nabla w\text{d}x=\int_{D}^{}F\cdot \nabla w\text{d}x\;\forall w\in H_{⋄}^{1}\left(\Omega \right). \end{array}$$

The energy term |∇*u*_0_|^2^ in Lemma 1 can be identified with the norm of the adjoint of this virtual measurement operator.

**Lemma 2**

The adjoint operator of *L*_*D*_ is given by

(17) $$\begin{array}{c} L_{D}^{*}:L_{⋄}^{2}\left(\Sigma \right)\to L^{2}{\left(D\right)}^{n},\;\;g\mapsto \nabla u_{0}{|}_{D}, \end{array}$$

where *u*_0_ ∈ *H*_⋄_^1^(*Ω*) solves

(18) $$\begin{array}{c} \nabla \cdot \sigma_{0}\nabla u_{0}=0\;in\;\;\Omega ,\;\;\sigma_{0}\partial_{\nu }u_{0}{|}_{\partial \Omega }=\{\begin{array}{cc} g & on\;\Sigma ,\\ 0 & else. \end{array} \end{array}$$

**Proof**

For all *g* ∈ *L*_⋄_^2^(Σ) and *F* ∈ *L* ^2^(*D*)^*n*^, we have that

(19) ∫D(LD∗g)·F dx=∫Σg(LDF)dx=∫Σgv|Σdx=∫Ωσ0∇u0·∇v dx=∫D∇u0·F dx,

which shows the assertion.

The following functional analytic lemma uses bounds on the image of an operator to characterize the range of its dual operator.

**Lemma 3**

Let *X* and *Y* be real Hilbert spaces with inner products (·, ·)_*X*_ and (·, ·)_*Y*_, respectively. Let *A* ∈ *ℒ*(*X*; *Y*) and *x*′ ∈ *X*. Then,

(20) x′∈ℛ(A∗) iff  ∃ C>0:|(x′,x)X|≤C||Ax|| ∀x∈X.

In particular, if *X*, *Y*_1_, and *Y*_2_ are three real Hilbert spaces, *A*_*i*_ ∈ *ℒ*(*Y*_*i*_, *X*), *i* = 1,2, and if there exists *C* > 0 with

(21) $$\begin{array}{c} ||A_{1}^{*}x||\leq C||A_{2}^{*}x||\;\forall x\in X, \end{array}$$

then *ℛ*(*A*_1_)⊆*ℛ*(*A*_2_).

**Proof**

The assertion can be generalized to Banach spaces, and, in that context, it is called the “14th important property of Banach spaces” in Bourbaki [26]. For the sake of completeness, we rewrite the proof from [27] to Hilbert spaces.

If *x*′ ∈ *ℛ*(A*), then there exists *y*′ ∈ *Y* such that *x*′ = A*y′. Hence,

(22) |(x′,x)X|=|(A∗y′,x)X|=|(y′,Ax)Y|≤||y′||||Ax|| ∀x∈X,

so that the assertion holds with *C* = ||*y*′||.

Now let *x*′ ∈ *X* be such that there exists *C* > 0 with |(*x*′, *x*)_*X*_ | ≤*C*||*Ax*|| for all *x* ∈ *X*. We define

(23) $$\begin{array}{c} f\left(y\right):={\left(x^{\prime },x\right)}_{X}\;\text{for}\;\text{every}\;\;y=Ax\in ℛ\left(A\right). \end{array}$$

Then, *f* is a well-defined, continuous linear functional on *ℛ*(*A*). By setting it to zero on *ℛ*(*A*)^⊥^, we can extend *f* to a continuous linear functional on *Y*. Using the Riesz theorem, it follows that there exists *y*′ ∈ *Y* with

(24) $$\begin{array}{c} {\left(y^{\prime },y\right)}_{Y}=f\left(y\right)\;\forall y\in ℛ\left(A\right). \end{array}$$

Hence, for all *x* ∈ *X*, we have

(25) $$\begin{array}{c} {\left(A^{*}y^{\prime },x\right)}_{X}={\left(y^{\prime },Ax\right)}_{Y}=f\left(Ax\right)={\left(x^{\prime },x\right)}_{X}, \end{array}$$

so that *x*′ = A*y′ ∈ *ℛ*(A*).

The last lemma shows that the range of the virtual measurement operator *L*_*D*_ determines the region *D* on which it is defined. We state the lemma for a simple special case, a generalized version of the lemma will be formulated in Section 3.2.

**Lemma 4**

Let *σ*_0_ = 1, *D*⊆*Ω* be open, and $\bar{D}\subseteq \Omega$ have a connected complement $\Omega \setminus \bar{D}$.

Then, for all unit vectors *d* ∈ ℝ^*n*^, ||*d*|| = 1, and every point *z* ∈ *Ω*∖∂*D*, it holds that

(26) $$\begin{array}{c} z\in D\;iff\;\;\Phi_{z,d}{|}_{\Sigma }\in ℛ\left(L_{D}\right). \end{array}$$

**Proof**

The proof is similar to the one of [21, Lemma 2.9].

First, let *z* ∈ *D* and *ϵ* > 0 be such that $\bar{B_{\epsilon }(z)}\subseteq D$. We choose

(27) $$\begin{array}{c} f_{1}\in H^{1}\left(B_{\epsilon }\left(z\right)\right)\;\text{with}\;f_{1}{|}_{\partial B_{\epsilon }(z)}=\Phi_{z,d}{|}_{\partial B_{\epsilon }(z)},\\ f_{2}\in H^{1}\left(B_{\epsilon }\left(z\right)\right)\;\text{with}\;\Delta f_{2}=0,\\ \partial_{\nu }f_{2}{|}_{\partial B_{\epsilon }(z)}=\partial_{\nu }\Phi_{z,d}{|}_{\partial B_{\epsilon }(z)}, \end{array}$$

and let *F* ∈ *L* ^2^(*D*)^*n*^ be the zero continuation of ∇(*f*_1_ − *f*_2_) to *D*.

Then, the function

(28) $$\begin{array}{c} v:=\{\begin{array}{cc} \Phi_{z,d} & \text{in}\;\Omega \setminus \bar{B_{\epsilon }\left(z\right)},\\ f_{1} & \text{in}\;B_{\epsilon }\left(z\right) \end{array} \end{array}$$

fulfills *v* ∈ *H*_⋄_^1^(*Ω*), and, for all *w* ∈ *H*_⋄_^1^(*Ω*),

(29) ∫Ω∇v·∇wdx =∫Ω∖Bϵ(z)¯∇Φz,d·∇wdx+∫Bϵ(z)∇f1·∇wdx =−∫∂Bϵ(z)∂νΦz,d  w|∂Bϵ(z)ds+∫Bϵ(z)∇f1·∇wdx =∫Bϵ(z)∇(f1−f2)·∇wdx=∫DF·∇wdx.

This shows that Φ_*z*,*d*_|_Σ_ = *v*|_Σ_ = *L*_*D*_(*F*) ∈ *ℛ*(*L*_*D*_).

Now let Φ_*z*,*d*_|_Σ_ ∈ *ℛ*(*L*_*D*_). Let *v* ∈ *H*_⋄_^1^(*Ω*) be the function from the definition of *L*_*D*_. Then,

(30) $$\begin{array}{c} v{|}_{\Sigma }=\Phi_{z,d}{|}_{\Sigma },\;\;\partial_{\nu }v{|}_{\Sigma }=0=\partial_{\nu }\Phi_{z,d}{|}_{\Sigma }, \end{array}$$

so that it follows by unique continuation that *v* = Φ_*z*,*d*_ in the connected set $\Omega \setminus (\bar{D}\cup \{z\})$.

If $z\notin \overset{-}{D}$, then $d\cdot \nabla \delta_{z}\notin H^{-2}(\Omega \setminus \bar{D})$, and thus $\Phi_{z,d}\notin L^{2}(\Omega \setminus \bar{D})$, which contradicts that *v* = Φ_*z*,*d*_ in $\Omega \setminus (\bar{D}\cup \{z\})$. Hence, $z\in \bar{D}$.

## 3. The Factorization Method

Now we will formulate the Factorization Method and characterize a region where a conductivity *σ* differs from a reference conductivity *σ*_0_ by a range criterion. Before we turn to a new general formulation of the method, we first state it for a special case that is similar to the one that was treated in the original works of Brühl and Hanke [3, 4].

### 3.1. The Factorization Method for a Simple Special Case

**Theorem 5**

Let *σ*_0_ = 1 and *σ* = 1 + *χ*_*D*_, where *D*⊆*Ω* is an open set so that $\bar{D}\subseteq \Omega$ has a connected complement $\Omega \setminus \bar{D}$. Then, for all *z* ∈ *Ω*, *z* ∉ ∂*D*, and all dipole directions *d* ∈ ℝ^*n*^, ||*d*|| = 1,

(31) $$\begin{array}{c} z\in D\;iff\;\;{\Phi_{z,d}|}_{\Sigma }\in ℛ\left({\left|\Lambda \left(\sigma \right)-\Lambda \left(1\right)\right|}^{1/2}\right). \end{array}$$

**Proof**

The monotony Lemma 1 yields that for all *g* ∈ *L*_⋄_^2^(∂*Ω*),

(32) $$\begin{array}{c} \int_{D}{\left|\nabla u_{0}\right|}^{2}\text{d}x\geq \int_{\Sigma }g\left(\Lambda \left(1\right)-\Lambda \left(\sigma \right)\right)g\text{d}s\geq \int_{D}\frac{1}{2}{\left|\nabla u_{0}\right|}^{2}\text{d}x. \end{array}$$

Hence, |Λ(*σ*) − Λ(1)| = Λ(1) − Λ(*σ*), and, using Lemma 2, we can restate this in the form

(33) $$\begin{array}{c} {||L_{D}^{*}g||}^{2}\geq {||{\left|\Lambda \left(\sigma \right)-\Lambda \left(1\right)\right|}^{1/2}g||}^{2}\geq \frac{1}{2}\;{||L_{D}^{*}g||}^{2}. \end{array}$$

Using the functional analytic Lemma 3, this implies that

(34) $$\begin{array}{c} ℛ\left(L_{D}\right)=ℛ\left({\left|\Lambda \left(\sigma \right)-\Lambda \left(1\right)\right|}^{1/2}\right), \end{array}$$

and thus the assertion follows from the relation between *D* and *ℛ*(*L*_*D*_) in Lemma 4.

Obviously, the same arguments can be used to treat the case *σ*(*x*) = 1 + *κ*(*x*)*χ*_*D*_(*x*), when there exists a conductivity jump *ϵ* > 0 so that either

(35) $$\begin{array}{c} \kappa \left(x\right)\geq \epsilon \;\forall x\in D\;\text{or}\;\kappa \left(x\right)\leq -\epsilon \;\forall x\in D. \end{array}$$

### 3.2. The Factorization Method for the General Piecewise Analytic Case

Now we drop the assumptions that the background is constant, that there is a clear conductivity jump, and that the complement of the inclusions is connected. We will merely assume that the reference conductivity *σ*_0_ is a piecewise analytic function and that either *σ* − *σ*_0_ ≥ 0 or *σ* − *σ*_0_ ≤ 0. Roughly speaking, under this general assumption, the Factorization Method then characterizes the support of *σ* − *σ*_0_ up to holes in the support that have no connections to Σ. For a precise formulation, we use the concept of the inner and outer support from [28] that has been inspired by the use of the infinity support of Kusiak and Sylvester [29]; see also [25, 30].

**Definition 6**

A relatively open set $U\subseteq \bar{\Omega }$ is called *connected* to Σ if *U*∩*Ω* is connected and *U*∩Σ ≠ *∅*.

For a measurable function *κ* : *Ω* → ℝ, we define

- the *support *supp⁡(*κ*) as the complement (in $\bar{\Omega }$) of the union of those relatively open $U\subseteq \bar{\Omega }$, for which *κ*|_*U*_ ≡ 0,
- the *inner support *inn supp⁡*κ* as the union of those open sets *U*⊆*Ω*, for which ess  inf_*x*∈*U*_ | *κ*(*x*)|>0,
- the *outer support *out_Σ_ supp⁡*κ* as the complement (in $\bar{\Omega }$) of the union of those relatively open $U\subseteq \bar{\Omega }$ that are connected to Σ and for which *κ*|_*U*_ ≡ 0.

The interior of a set *M*⊆*Ω* is denoted by int⁡*M* and its closure (with respect to ℝ^*n*^) by $\bar{M}$. If *M* is measurable, we also define

(d) out_Σ_ *M* = out_Σ_supp⁡*χ*_*M*_.

It is easily checked that out_Σ_(supp⁡*κ*) = out_Σ_supp⁡*κ*.

With this concept, we can extend the range characterization in Lemma 4 to a general setting (see also Remark 9 later).

**Lemma 7**

Let *σ*_0_ ∈ *L*_+_^*∞*^(*Ω*) be piecewise analytic. Let *D*⊆*Ω* be measurable.

Then, for all unit vectors *d* ∈ ℝ^*n*^, ||*d*|| = 1, and every point *z* ∈ *Ω* that has a neighborhood in which *σ*_0_ is analytic,

(36) $$\begin{array}{c} z\in \mathrm{int}D\;implies\;\;{\Phi_{z,d}|}_{\Sigma }\in ℛ\left(L_{D}\right), \end{array}$$

and

(37) $$\begin{array}{c} {\Phi_{z,d}|}_{\Sigma }\in ℛ\left(L_{D}\right)\;implies\;\;z\in {\text{out}}_{\Sigma }D. \end{array}$$

**Proof**

If *z* ∈ int⁡*D*, then there exists a small ball $\bar{B_{\epsilon }(z)}\subseteq D$, and the first assertion follows as in the proof of Lemma 4.

To show the second assertion, let Φ_*z*,*d*_|_Σ_ ∈ *ℛ*(*L*_*D*_), and let *v* ∈ *H*_⋄_^1^(*Ω*) be the function from the definition of *L*_*D*_, so that (as in the proof of Lemma 4)

(38) $$\begin{array}{c} v{|}_{\Sigma }=\Phi_{z,d}{|}_{\Sigma },\;\;\partial_{\nu }v{|}_{\Sigma }=0=\partial_{\nu }\Phi_{z,d}{|}_{\Sigma }. \end{array}$$

Assume that *z* ∉ out_Σ_ *D*. Then, there exists a relatively open $U\subseteq \bar{\Omega }$ that is connected to Σ and contains *z*. Hence, by unique continuation, it follows that *v*|_*U*_ = Φ_*z*,*d*_|_*U*_, and we obtain the same contradiction as in the proof of Lemma 4.

Now, we can formulate and prove the Factorization Method for general piecewise analytic conductivities.

**Theorem 8**

Let *σ* ∈ *L*_+_^*∞*^(*Ω*), and let *σ*_0_ ∈ *L*_+_^*∞*^(*Ω*) be a piecewise analytic function. Let either

(39) $$\begin{array}{c} \sigma \left(x\right)\geq \sigma_{0}\left(x\right)\;\forall x\in \Omega \;\text{or}\;\sigma \left(x\right)\leq \sigma_{0}\left(x\right)\;\forall x\in \Omega . \end{array}$$

Then, for all *z* ∈ *Ω* that have a neighborhood in which *σ*_0_ is analytic, as well as all unit vectors *d* ∈ ℝ^*n*^, ||*d*|| = 1,

(40) z∈
inn supp
 (σ−σ0) implies  Φz,d|Σ∈ℛ(|Λ(σ)−Λ(σ0)|1/2),

and

(41) Φz,d|Σ∈ℛ(|Λ(σ)−Λ(σ0)|1/2) implies  z∈outΣsupp⁡(σ−σ0).

**Proof**

Let *z* ∈ *Ω* have a neighborhood in which *σ*_0_ is analytic, and let *d* ∈ ℝ^*n*^ be a unit vector with ||*d*|| = 1. We only prove the assertions for *σ* ≥ *σ*_0_. The other case is completely analogous.

First, let *z* ∈ inn supp⁡  (*σ* − *σ*_0_). Then there exists a small ball *B*_*ϵ*_(*z*) and *δ* > 0 so that *σ* − *σ*_0_ ≥ *δ* on *B*_*ϵ*_(*z*). Using the monotony Lemma 1, it follows that, for all *g* ∈ *L*_⋄_^2^(∂*Ω*),

(42) ∫Σg(Λ(σ0)−Λ(σ))gds ≥∫Ωσ0σ(σ−σ0)|∇u0|2dx ≥δ||σ0||L∞(Ω)||1σ||L∞(Ω)∫Bϵ(z)|∇u0|2dx ≥δ||σ0||L∞(Ω)||1σ||L∞(Ω)||LBϵ(z)∗g||2.

Using the functional analytic Lemma 3, we obtain that

(43) $$\begin{array}{c} ℛ\left(L_{B_{\epsilon }(z)}\right)\subseteq ℛ\left({\left|\Lambda \left(\sigma \right)-\Lambda \left(\sigma_{0}\right)\right|}^{1/2}\right), \end{array}$$

and Lemma 7 yields that

(44) $$\begin{array}{c} \Phi_{z,d}{|}_{\Sigma }\in ℛ\left(L_{B_{\epsilon }(z)}\right)\subseteq ℛ\left({\left|\Lambda \left(\sigma \right)-\Lambda \left(\sigma_{0}\right)\right|}^{1/2}\right). \end{array}$$

On the other hand, with *D* : = supp⁡(*σ* − *σ*_0_), the monotony Lemma 1 shows that for all *g* ∈ *L*_⋄_^2^(∂*Ω*),

(45) ∫Σg(Λ(σ0)−Λ(σ1))gds≤∫Ω(σ1−σ0)|∇u0|2dx≤  ||σ1−σ0||L∞(Ω)∫D|∇u0|2dx≤  ||σ1−σ0||L∞(Ω)||LD∗g||2,

so that we obtain from the functional analytic Lemma 3

(46) $$\begin{array}{c} ℛ\left({\left|\Lambda \left(\sigma \right)-\Lambda \left(\sigma_{0}\right)\right|}^{1/2}\right)\subseteq ℛ\left(L_{D}\right). \end{array}$$

Hence, Lemma 7 yields that

(47) Φz,d|Σ∈ℛ(|Λ(σ)−Λ(σ0)|1/2)⊆ℛ(LD)  implies  z∈outΣ (supp⁡(σ−σ0))=outΣ supp⁡  (σ−σ0).

**Remark 9**

Theorem 8 shows that the Factorization Method is able to detect the support of a conductivity difference up to the difference between the outer and the inner support, that is, roughly speaking, up to holes in the support that have no connections to the boundary. It leaves open whether points in such holes will fulfill the range criterion of the Factorization Method or not.

A result of Hyvönen and the author [30, Lemma 2.5] shows that for every smooth domain *D* with $\bar{D}\subset \Omega$ and every unit vector *d* ∈ ℝ^*n*^, ||*d*|| = 1,

(48) Φz,d|Σ∈ℛ(|Λ(σ)−Λ(σ0)|1/2) ∀z∈∂D

implies

(49) $$\begin{array}{c} \Phi_{z,d}{|}_{\Sigma }\in ℛ\left({\left|\Lambda \left(\sigma \right)-\Lambda \left(\sigma_{0}\right)\right|}^{1/2}\right)\;\forall z\in D. \end{array}$$

In that sense, we can expect that holes in the support will be filled up and that the set detected by the Factorization Method is essentially the outer support of the conductivity difference.

### 3.3. The Factorization Method for the Indefinite Case

It is a long standing open theoretical problem whether the range criterion of the Factorization Method holds true without the *definiteness assumption* that *σ* ≥ *σ*_0_ on *Ω* or *σ* ≤ *σ*_0_ on *Ω*. However, Grinberg, Kirsch, and Schmitt [18, 31] showed how to *exclude* a region *E*⊆*Ω* from *Ω*, in such a way that the Factorization Method only requires the definiteness assumption on *Ω*∖*E*. In this subsection, we show how their idea can be incorporated into our formulation of the method.

To point out the main idea, we first formulate the result for a simple special case. Let us stress that, for *σ* = 1 + *χ*_*D*^+^_ − (1/2)*χ*_*D*^−^_, it is not known whether

(50) z∈D+∪D− iff  Φz,d|Σ∈ℛ(|Λ(σ)−Λ(1)|1/2).

However, we can still use the Factorization Method if we have some a priori knowledge that separates *D* ^+^ and *D* ^−^. More precisely, if we know a subset *E* that contains *D* ^−^ without intersecting *D* ^+^, then we can use the Factorization Method to find *D* ^+^ ∪ *E* (and thus *D* ^+^).

**Theorem 10**

Let *σ*_0_ = 1 and *σ* = 1 + *χ*_*D*^+^_ − (1/2)*χ*_*D*^−^_, where *D* ^+^, *D* ^−^⊆*Ω* are open. Let *E*⊆*Ω* be an open set.

(a) If *D* ^+^⊆*E* and $\bar{D^{-}\cup E}\subseteq \Omega$ has a connected complement, then for all *z* ∈ *Ω*, *z* ∉ ∂(*D* ^−^ ∪ *E*), and all dipole directions *d* ∈ ℝ^*n*^, ||*d*|| = 1,
  (51) z∈D−∪E iff  Φz,d|Σ∈ℛ(|Λ(σ)−Λ(1)+LELE∗|1/2).
(b) If *D* ^−^⊆*E* and $\bar{D^{+}\cup E}\subseteq \Omega$ has a connected complement, then for all *z* ∈ *Ω*, *z* ∉ ∂(*D* ^+^ ∪ *E*), and all dipole directions *d* ∈ ℝ^*n*^, ||*d*|| = 1,
  (52) z∈D+∪E iff  Φz,d|Σ∈ℛ(|Λ(σ)−Λ(1)−LELE∗|1/2).

**Proof**

The monotony Lemma 1 yields that for all *g* ∈ *L*_⋄_^2^(∂*Ω*),

(53) 12∫D−|∇u0|2dx−∫D+|∇u0|2dx ≤∫Σg(Λ(σ)−Λ(1))gds ≤∫D−|∇u0|2dx−12∫D+|∇u0|2dx.

Since (cf. Lemma 2)

(54) $$\begin{array}{c} \int_{\Sigma }g\left(L_{E}L_{E}^{*}\right)g\text{d}s={||L_{E}^{*}g||}^{2}=\int_{E}{\left|\nabla u_{0}\right|}^{2}\text{d}x, \end{array}$$

it follows for case (a) that

(55) 12∫D−∪E|∇u0|2dx≤∫Σg(Λ(σ)−Λ(1)+2LELE∗)gds≤∫D−∪E|∇u0|2dx.

Using the functional analytic Lemma 3, this implies that

(56) $$\begin{array}{c} ℛ\left(L_{D^{-}\cup E}\right)=ℛ\left({\left|\Lambda \left(\sigma \right)-\Lambda \left(1\right)+2L_{E}L_{E}^{*}\right|}^{1/2}\right), \end{array}$$

so that the assertion (a) follows from Lemma 4.

In case (b), we obtain that

(57) 32∫D+∪E|∇u0|2dx≥∫Σg(Λ(1)−Λ(σ)+2LELE∗)gds≥12∫D+∪E|∇u0|2dx,

and the same arguments as above yield the assertion.

We can also extend these ideas to the general setting of Section 3.2.

**Theorem 11**

Let *σ* ∈ *L*_+_^*∞*^(*Ω*) and let *σ*_0_ ∈ *L*_+_^*∞*^(*Ω*) be a piecewise analytic function. Let *E*⊆*Ω* be a measurable set.

Choose *α*, *β* ∈ ℝ such that

(58) $$\begin{array}{c} \alpha >\;{||\sigma -\sigma_{0}||}_{L^{\infty }(\Omega )},\;\;\beta >{||\frac{\sigma_{0}}{\sigma }\left(\sigma_{0}-\sigma \right)||}_{L^{\infty }(\Omega )}. \end{array}$$

(a) If *σ* ≤ *σ*_0_ on *Ω*∖*E*, then for all *z* ∈ *Ω* that have a neighborhood in which *σ*_0_ is analytic, as well as all unit vectors *d* ∈ ℝ^*n*^, ||*d*|| = 1,
  (59) z∈ 
inn supp
 (σ−σ0)∪E  implies  Φz,d|Σ∈ℛ(|Λ(σ)−Λ(σ0)+αLELE∗|1/2),
 and
  (60) Φz,d|Σ∈ℛ(|Λ(σ)−Λ(σ0)+αLELE∗|1/2)  implies  z∈outΣ(supp⁡(σ−σ0)∪E).
(b) If *σ* ≥ *σ*_0_ on *Ω*∖*E*, then for all *z* ∈ *Ω* that have a neighborhood in which *σ*_0_ is analytic, as well as all unit vectors *d* ∈ ℝ^*n*^, ||*d*|| = 1,
  (61) z∈ 
inn supp
(σ−σ0)∪E,  implies  Φz,d|Σ∈ℛ(|Λ(σ)−Λ(σ0)−βLELE∗|1/2)
 and
  (62) Φz,d|Σ∈ℛ(|Λ(σ)−Λ(σ0)−βLELE∗|1/2)  implies  z∈outΣ(supp⁡(σ−σ0)∪E).

**Proof**

For every *z* ∈ inn supp⁡(*σ* − *σ*_0_) with *z* ∉ *E*, there exists a small ball *B*_*ϵ*_(*z*) and *δ* > 0 so that *σ*_0_ − *σ* ≥ *δ* on *B*_*ϵ*_(*z*). Using the monotony Lemma 1, it follows that, for all *g* ∈ *L*_⋄_^2^(∂*Ω*),

(63) ∫Σg(Λ(σ)−Λ(σ0)+αLELE∗)gds ≥∫Ω(σ0−σ1)|∇u0|2dx+α∫E|∇u0|2dx ≥δ∫Bϵ(z)|∇u0|2dx+(α−  ||σ−σ0||L∞(Ω))∫E|∇u0|2dx.

As in the previous proofs, we obtain from Lemmas 2 and 3 that

(64) $$\begin{array}{c} ℛ\left({\left|\Lambda \left(\sigma \right)-\Lambda \left(\sigma_{0}\right)+\alpha L_{E}L_{E}^{*}\right|}^{1/2}\right)⊇ℛ\left(L_{B_{\epsilon }(z)\cup E}\right), \end{array}$$

so that the first implication of (a) follows from Lemma 7.

The monotony Lemma 1 also implies that

(65) ∫Σg(Λ(σ)−Λ(σ0)+αLELE∗)gds ≤∫Ωσ0σ(σ0−σ)|∇u0|2dx+α∫E|∇u0|2dx ≤||σ0σ(σ0−σ)||L∞(Ω)∫supp⁡(σ−σ0)|∇u0|2dx  +α∫E|∇u0|2dx,

so that the second implication of (a) follows from Lemmas 2, 3, and 7. Assertion (b) can be proven analogously.

Remark 9 also applies to this case.

## 4. Conclusions and Remarks

 The Factorization Method can be used to detect regions in which a conductivity differs from a known reference conductivity. In this work, we summarized the progress on the method's theoretical foundation. We formulated the method for general piecewise analytic conductivities and gave comparatively simple and self-contained proofs. We also showed how the idea of excluding a part of the imaging region can be incorporated into this formulation.

The regularity assumptions can be weakened even further. Our proofs only require unique continuation arguments for the reference conductivity *σ*_0_ and the existence of the dipole functions.

Two major open theoretical questions still exist in the context of the Factorization Method. The theoretical justification of the method requires a definiteness condition (on the whole domain or after excluding an a priori known part of the domain). It is unknown whether the method's range criterion holds without such a definiteness condition. The second open question concerns the numerical stability of the method's range criterion. So far, there are no rigorous convergence results for numerical implementations of this range criterion (see, however, Lechleiter [32] for a first step in this direction). As a promising approach to overcome both problems, we would like to point out the recent work on monotony-based methods [28].
